# Neighborhood Characteristics and Elevated Blood Pressure in Older Adults

**DOI:** 10.1001/jamanetworkopen.2023.35534

**Published:** 2023-09-25

**Authors:** Kendra D. Sims, Mary D. Willis, Perry W. Hystad, G. David Batty, Kirsten Bibbins-Domingo, Ellen Smit, Michelle C. Odden

**Affiliations:** 1 Department of Epidemiology and Biostatistics, University of California, San Francisco; 2 Department of Epidemiology, Boston University School of Public Health, Boston, Massachusetts; 3 School of Biological and Population Health Sciences, College of Public Health and Human Sciences, Oregon State University, Corvallis; 4 Department of Epidemiology and Public Health, University College London, London, United Kingdom; 5 Department of Epidemiology and Population Health, Stanford University School of Medicine, Stanford, California; 6 Editor in Chief, *JAMA*

## Abstract

**Importance:**

The local environment remains an understudied contributor to elevated blood pressure among older adults. Untargeted approaches can identify neighborhood conditions interrelated with racial segregation that drive hypertension disparities.

**Objective:**

To evaluate independent associations of sociodemographic, economic, and housing neighborhood factors with elevated blood pressure.

**Design, Setting, and Participants:**

In this cohort study, the sample included Health and Retirement Study participants who had between 1 and 3 sets of biennial sphygmomanometer readings from 2006 to 2014 or 2008 to 2016. Statistical analyses were conducted from February 5 to November 30, 2021.

**Exposures:**

Fifty-one standardized American Community Survey census tract variables (2005-2009).

**Main Outcomes and Measures:**

Elevated sphygmomanometer readings over the study period (6-year period prevalence): a value of at least 140 mm Hg for systolic blood pressure and/or at least 90 mm Hg for diastolic blood pressure. Participants were divided 50:50 into training and test data sets. Generalized estimating equations were used to summarize multivariable associations between each neighborhood variable and the period prevalence of elevated blood pressure, adjusting for individual-level covariates. Any neighborhood factor associated (Simes-adjusted for multiple comparisons *P* ≤ .05) with elevated blood pressure in the training data set was rerun in the test data set to gauge model performance. Lastly, in the full cohort, race- and ethnicity-stratified associations were evaluated for each identified neighborhood factor on the likelihood of elevated blood pressure.

**Results:**

Of 12 946 participants, 4565 (35%) had elevated sphygmomanometer readings (median [IQR] age, 68 [63-73] years; 2283 [50%] male; 228 [5%] Hispanic or Latino, 502 [11%] non-Hispanic Black, and 3761 [82%] non-Hispanic White). Between 2006 and 2016, a lower likelihood of elevated blood pressure was observed (relative risk for highest vs lowest tertile, 0.91; 95% CI, 0.86-0.96) among participants residing in a neighborhood with recent (post-1999) in-migration of homeowners. This association was precise among participants with non-Hispanic White and other race and ethnicity (relative risk, 0.91; 95% CI, 0.85-0.97) but not non-Hispanic Black participants (relative risk, 0.97; 95% CI, 0.85-1.11; *P* = .48 for interaction) or Hispanic or Latino participants (relative risk, 0.84; 95% CI, 0.65-1.09; *P* = .78 for interaction).

**Conclusions and Relevance:**

In this cohort study of older adults, recent relocation of homeowners to a neighborhood was robustly associated with reduced likelihood of elevated blood pressure among White participants but not their racially and ethnically marginalized counterparts. Our findings indicate that gentrification may influence later-life blood pressure control.

## Key Points

**Question:**

Which sociodemographic, economic, and housing neighborhood factors are associated with elevated blood pressure among older adults?

**Findings:**

In this cohort study of a nationwide sample of 12 946 adults older than 50 years, 1 of 51 evaluated census tract factors had statistically significant associations with blood pressure control after adjustment for multiple comparisons and individual-level confounders. Residing in a census tract experiencing the highest vs lowest tertile of post-1999 in-migration of homeowners was associated with reduced relative risk of elevated blood pressure from 2006 to 2016; this association was only present among non-Hispanic White participants.

**Meaning:**

Gentrification via in-migration of homeowners may influence later-life blood pressure control.

## Teaser

This cohort study examines data from Health and Retirement Study participants to determine associations between blood pressure control and sociodemographic, economic, and housing factors at the census tract level.

## Introduction

The neighborhood environment in which older adults spend increasing amounts of their time after retirement remains an understudied contributor to elevated blood pressure,^1,2,3,4,5,6^ a leading modifiable risk factor for cardiovascular disease (CVD), disability, and mortality.^7,8^ Racial segregation,^9,10^ suppressed local economies,^11,12,13,14^ and crowded, dilapidated housing conditions^15,16,17^ have been associated with increased likelihood of hypertension. These neighborhood factors may differentially influence CVD risk for socially dominant vs marginalized groups. Insufficient investment in the local infrastructure likely leaves residents who have low socioeconomic status (SES) and identify as racial and ethnic minorities without the social or financial resources to manage elevated blood pressure.^18^

**Figure 1.**
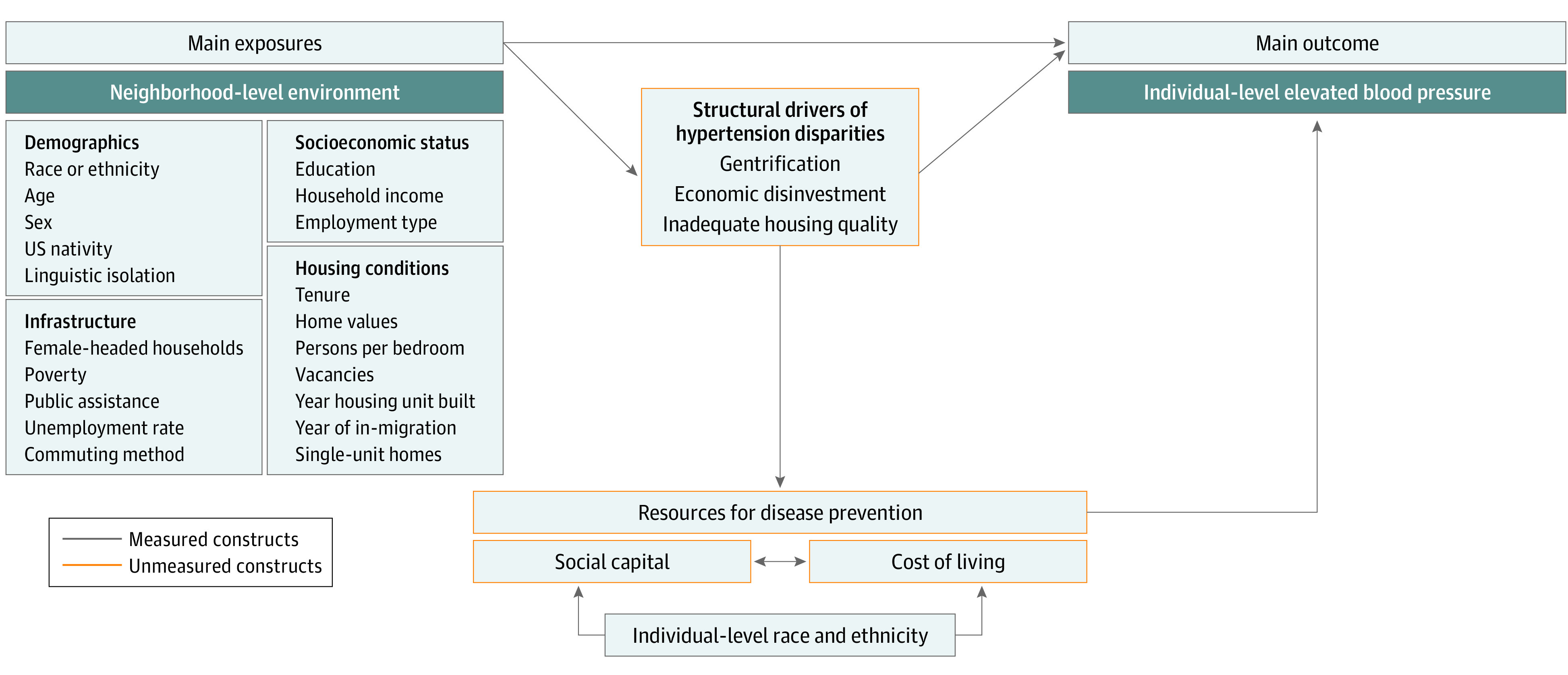
Hypothesized Pathways Between Neighborhood Environment and Racial and Ethnic Disparities in Elevated Blood Pressure Among Older Adults

Revitalization via gentrification is framed as a solution for the adverse environmental context experienced by marginalized people.^19,20^ However, the increasing property values and new amenities that promote in-migration of residents with high SES may increase the cost of living in the gentrified area to the point that existing residents cannot afford lifestyles that preserve CVD health.^20,21^ As racial and ethnic minority adults seem to receive diminishing returns from the health-protecting benefits of higher education and income,^22^ the apparently opposing processes of disinvestment and reinvestment via gentrification may similarly act as structural drivers of older adult hypertension disparities^23^ (Figure 1).

As the co-occurrence of adverse neighborhood conditions is commonly not accounted for in analyses, remaining challenges to neighborhood inequity research include parsing the independent association of area demographics (ie, racial segregation) from indicators of disinvested local economies, crowded or older housing conditions, and gentrification. Furthermore, although individual and area-level SES are at least moderately correlated, failure to sufficiently control for individual-level social determinants of health when analyzing the health-related impact of adverse neighborhood context may systematically bias findings among older, low-income, and rural residents most vulnerable to hypertension and adverse sequalae.^24,25^ Neighborhood-wide association studies are a methodological strategy to account for the probability of incorrectly rejected null hypotheses across multiple comparisons (ie, the false discovery rate) of interrelated neighborhood conditions while controlling for a consistent set of individual-level confounders.^26,27^ Untargeted approaches such as these are vital to identify adverse economic and housing conditions, which often occur in racially segregated neighborhoods and correspond with an excess population-level burden of hypertension.

These research gaps inform this neighborhood-wide association study of factors associated with risk of elevated blood pressure among middle-aged and older adults. By assessing independent associations of neighborhood factors as determinants of elevated blood pressure, we build on previous work evaluating interpersonal discrimination and hypertensive outcomes in a nationally representative sample of US middle-aged and older adults.^28,29^ We evaluated whether residence in neighborhoods with indicators of economic disinvestment, gentrification, or inadequate housing quality was associated with a higher relative risk of elevated blood pressure after adjustment for multiple comparisons and individual-level covariates. We also assessed whether these associations differed by race and ethnicity.

## Methods

### Study Sample

The Health and Retirement Study (HRS) collects comprehensive socioeconomic and well-being information to ascertain the dynamics of the aging process.^30^ This cohort is intended to be representative of the initially noninstitutionalized population older than 50 years. A 4-stage sampling strategy provides probabilistic coverage of metropolitan statistical areas or counties located within the contiguous 48 states; census tracts in Florida, as well as those with a high proportion of Black and Hispanic residents, were overrepresented by approximately 2-fold. HRS provides documentation on informed consent and requires verbal consent for participation; the University of Michigan institutional review board approved the parent study protocol. The present data management and analyses were exempt from additional review by the institutional review board because we used deidentified data with area-level linkages no more granular than the census tract. We followed the Strengthening the Reporting of Observational Studies in Epidemiology (STROBE) reporting guideline for cohort studies.

Assignment to in-person and telephonic interviews alternated every 2 years. Half the sample was eligible for an in-person home interview in 2006, contributing sphygmomanometer readings during physical functioning measurements.^31^ The other half of the sample completed telephone interviews and was assigned to a 2008 face-to-face interview. After the interview, participants self-completed a questionnaire regarding marital status and health. We concatenated these halves of the sample for this period prevalence analysis, where blood pressure was measured at 3 points: either 2006, 2010, and 2014 or 2008, 2012, and 2016. Participants needed an address linked to census data and complete covariate data (N = 12 946) (eFigure in Supplement 1).

### Outcomes

Using Omron HEM-780 Intellisense automated sphygmomanometers with ComFit cuffs, trained interviewers biennially took 3 blood pressure readings from seated participants who had both feet on the floor.^31^ We used the mean of 3 systolic and diastolic readings at each visit, taken between 45 and 60 seconds apart from a participant’s supported left arm with palm upturned. Mean blood pressure readings below 40 mm Hg (n = 142) were excluded because of probable measurement error.

Elevated blood pressure was classified according to the Seventh Report of the Joint National Committee on Prevention, Detection, Evaluation, and Treatment of High Blood Pressure (JNC 7) criteria: at least 140 mm Hg for systolic and/or at least 90 mm Hg for diastolic blood pressure.^32^ The JNC 7 were the blood pressure control guidelines known to health care professionals and study participants between 2006 and 2016.

### Individual-Level Covariates

We adjusted for the baseline self-reported social determinants of health that we identified as relevant in prior work.^28^ Besides age in years, we accounted for sex to approximate gender, highest educational attainment (less than high school, high school degree or General Educational Development test achievement [GED], 2-year college degree, at least a 4-year college degree), US nativity (yes or no), marital or partnership status (married/partnered, divorced/separated, widowed, never married/partnered), type of insurance coverage (private, only federal/public, neither public or private), home ownership (yes or no), and household net income compared with the Current Population Survey 2000 poverty threshold (above, below, income not reported). We used the following categories of self-reported racial and ethnic designation: Hispanic or Latino, non-Hispanic Black, non-Hispanic White, and other. Other consisted of participants reporting American Indian or Alaska Native, Asian or Pacific Islander, or “don’t know” and those who chose not to report. We only had adequate sample size to compare the association of neighborhood factors with Hispanic or Latino, non-Hispanic Black, and non-Hispanic White older adults. Estimates and precision did not appreciably change across analyses that excluded the 208 other participants and analyses that grouped data for other with those for non-Hispanic White; we presented the latter set of estimates. To account for temporal trends, we also included a fixed effect for a baseline study inclusion wave of 2006 vs 2008.

### Neighborhood-Level Exposures

Our evaluated exposures come from the 2005-2009 American Community Survey (ACS), conducted by the Census Bureau via annual random samples of housing units.^33^ HRS provides researchers a managed set of select ACS variables via a remote data enclave to maintain participant anonymity. We used factors at the level of census tract to approximate neighborhoods. ACS annual data are aggregated into 5-year chunks for more robust estimates and can be considered a period estimate of assorted census tract characteristics between 2005 and 2009. Because of temporal changes to geographic units, ACS interpolated annual census tract boundaries between years. We presented the differences in census tract factors by participant baseline hypertensive status in the eTable (Supplement 1).

#### Demographic

The age distribution was assessed with 3 proportional variables: younger than 18 years, between 18 and 64 years, and at least 65 years old. The proportions of males and females at least 65 years old were also evaluated.

#### Racial and Ethnic Composition

The proportions of reported racial and ethnic identities we assessed include Hispanic or Latino, non-Hispanic Asian, non-Hispanic Black, non-Hispanic White, and non-Hispanic other or multiple. The proportions of individuals born in the United States, speaking English only at home, speaking English well at home, and household linguistic isolation (ie, all adults in the household speak a language other than English and none speak English very well) were included to assess immigration patterning and acculturation.

#### Socioeconomic Status

Proportional variables assessing educational attainment included having no high school diploma, at least a high school diploma or equivalent (GED), and a college degree. Household-level factors relevant to area deprivation included the proportions of female-headed households, income falling below the poverty line, and receiving public-assistance income.

#### Labor Force Factors

Proportional factors related to occupational status included workforce participation, unemployment rate, employment in management and professional occupations, and employment in the manufacturing industry. Main commuting mode among workers at least 16 years old was determined with variables designating proportions who drove, took public transit, and walked or biked.

#### Housing

Proportional variables related to housing conditions included living alone (in the full population, as well as among the subgroups of those at least 65 years old, male, and female), home ownership, vacant homes for sale or rent, homes with more than 1 person per room, year built for housing units (before 1980, from 1980 to 1999, and after 1999), year moved for owners and renters, respectively (after 1999 or after 2004), and single-unit homes. Continuous median values for house value, house value for owner-occupied housing units, and gross rent were also evaluated.

### Statistical Analysis

**Figure 2.**
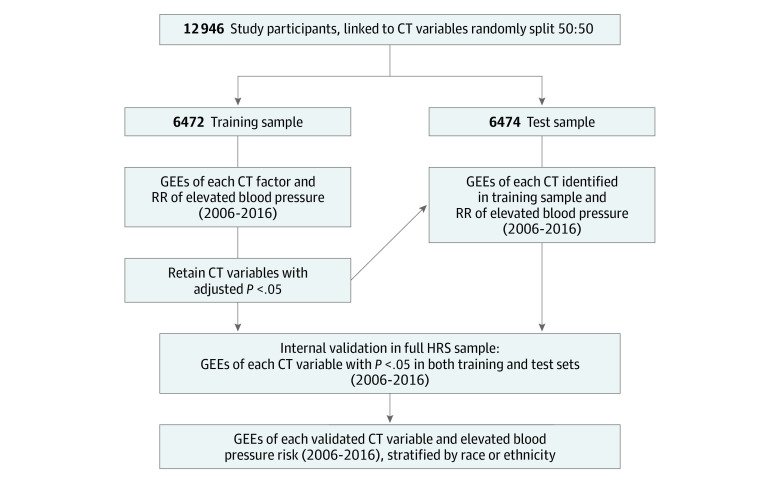
Analytic Process for Neighborhood-Wide Association Study of Elevated Blood Pressure CT indicates census tract; GEEs, generalized estimating equations; HRS, Health and Retirement Study; RR, relative risk.

We first described individual characteristics of the sample at either 2006 or 2008 across hypertensive status (normotensive and hypertensive). Based on previous neighborhood-wide association studies,^19,21,25^ we conducted a multistage method similar to a genome-wide association study (Figure 2). We accounted for skew in age, population density, area-level income, rental prices, and housing values by log-transforming these variables in all analyses. To obtain comparable estimates across multiple comparisons, we *z* score–standardized all census tract variables. We removed census tract factors that were collinear (*r* < 0.99) with other factors from evaluation as potential exposures. We randomly divided participants into training (n = 6472) and test (n = 6474) samples.

To identify independent associations across potential exposures, we first evaluated each census tract factor in parallel multivariable models among the training set with generalized estimating equations to estimate associations between each census tract factor on the likelihood of elevated blood pressure between 2006 and 2008 and between 2014 and 2016. Because individual-level hypertensive status did not significantly change between the 3 waves in the sample, we estimated the 6-year mean period prevalence of elevated blood pressure across 3 sets of biennial sphygmomanometer readings. We specified a logistic link function, binomial distribution, robust standard error, and independent correlation structure. We applied the Simes adjustment of the expected proportion of false positives out of all the rejected null hypotheses observed across these simultaneous comparisons (ie, the false discovery rate).^34^ The false discovery rate grants more statistical power than the more conservative family-wise error rate, because the latter controls for the probability of making at least 1 false discovery and therefore increases the likelihood of making false negatives.^35^ To account for findings attributable to random fluctuations in the training sample, we then reran the model with the neighborhood factor identified in the training sample as significant (*P* < .05) in the other half of the sample as a test for replication. A census tract factor that was significant in both halves of the sample was considered a consistent or internally validated finding.

We presented estimates with tertiles of census tract factors to increase estimate interpretability. We fit interaction terms between each validated factor and racial and ethnic designation to evaluate differences between participants who were non-Hispanic White or other, non-Hispanic Black, and Hispanic or Latino. We conducted all analyses using Stata version 17.1 (StataCorp). To account for the complex sampling methodology of HRS, all estimates were weighted by the 2006 or 2008 baseline probability of being eligible for parent study inclusion. Statistical analyses were conducted from February 5 to November 30, 2021.

## Results

Thirty-five percent of the sample of 12 946 adults (aged 51-101 years; 11 002 [85%] non-Hispanic White) had hypertensive sphygmomanometer readings in either 2006 or 2008. Compared with normotensive participants, those with elevated blood pressure according to JNC 7 criteria were older (median [IQR] age, 65 [60-75] vs 68 [63-73] years), were more likely to be male (3520 [42%] vs 2283 [50%]), and more frequently identified as non-Hispanic Black (587 [7%] vs 502 [11%]). Participants with elevated blood pressure at baseline were less likely those with normotensive sphygmomanometer readings to have attained a 4-year college degree (959 [21%] vs 2263 [27%]), to be currently married (2739 [60%] vs 5531 [66%]), to have private insurance coverage (2922 [64%] vs 5783 [69%]), or to own their homes (2602 [57%] vs 5112 [61%]) (Table 1). Unadjusted differences in census tract–level sociodemographic, economic, and housing factors by 2006-2008 hypertensive status overall differed by less than 1% (eTable in Supplement 1).

**Table 1.** Baseline Individual Characteristics of Health and Retirement Study Participants, Aged 51 to 101 Years, by Hypertensive Status, 2006-2008 (N = 12 946)

Characteristic	Participants, No. (%)		
	Normotensive (n = 8381)	Hypertensive (n = 4565)	*P* value^a^
Age, median (IQR), y	65 (60-75)	68 (63-73)	<.001
Sex			
Male	3520 (42)	2283 (50)	<.001
Female	4861 (58)	2282 (50)	
Racial and ethnic designation			
Hispanic or Latino	419 (5)	228 (5)	<.001
Non-Hispanic Black	587 (7)	502 (11)	
Non-Hispanic White	7241 (86)	3761 (82)	
Other^b^	134 (2)	74 (2)	
US nativity	7711 (92)	4109 (90)	<.001
Marital/partnership status			
Married/partnered	5531 (66)	2739 (60)	<.001
Divorced/separated	1257 (15)	730 (16)	
Widowed	1257 (15)	913 (20)	
Never married/partnered	335 (4)	183 (4)	
Highest educational attainment			
Less than high school degree	1257 (15)	959 (21)	<.001
High school degree/GED	4442 (53)	2465 (54)	
2-y College degree	419 (5)	183 (4)	
At least 4-y college degree	2263 (27)	959 (21)	
Insurance type^c^			
Private	5783 (69)	2922 (64)	<.001
Only federal/public	2095 (25)	1370 (30)	
Neither public or private	503 (6)	274 (6)	
Owns home	5112 (61)	2602 (57)	<.001
Household poverty status^d^			
Income above federal poverty line	4526 (54)	2511 (55)	.31
Income below federal poverty line	335 (4)	183 (4)	
Unknown income	3520 (42)	1872 (41)	

Abbreviation: GED, General Educational Development test achievement.

^a^ *P* value (Pearson χ^2^ test for categorical variables, Wilcoxon rank sum test for continuous variables) for difference between normotensive vs hypertensive sphygmomanometer reading.

^b^ Participants reporting that they self-identified as American Indian or Alaska Native, Asian or Pacific Islander, “don’t know,” or “refused.”

^c^ Reported private insurance indicates employer-based or individually purchased, while federal/public indicates Medicare, Medicaid, Civilian Health and Medical Program Uniformed Service, and Department of Veterans Affairs.

^d^ Household net income classifications are relative to the poverty line as determined by the 2000 Current Population Survey.

We removed the proportion of females and the proportions of males at least 65 years old living alone because of collinearity with other census tract factors. After adjustment for individual factors as well as well as multiple comparisons, 3 of 51 census tract factors were independently associated with elevated blood pressure in the training set (Table 2). Participants living in census tracts with a higher proportion of housing units built before 1980 had a higher likelihood of elevated blood pressure in the training set (RR for highest vs lowest tertile, 1.12; 95% CI, 1.03-1.22), although results were attenuated in the test set (RR for highest vs lowest tertile, 1.01; 95% CI, 0.93-1.10). Similarly, participants in census tracts with the highest proportion of renters moving in after 1999 appeared to be associated with a lower likelihood of elevated blood pressure in the training set (RR for highest vs lowest tertile, 0.89; 95% CI, 0.82-0.96) but not in the test set (RR for highest vs lowest tertile, 0.97; 95% CI, 0.90-1.05). We observed a consistent association between a higher proportion of homeowners who moved into the census tract after 1999 in the training set (RR for highest vs lowest tertile, 0.90; 95% CI, 0.81-0.95) and test set (RR for highest vs lowest tertile, 0.93; 95% CI, 0.85-1.00). In the full sample, more recent in-migration of homeowners was associated with a lower time-averaged risk of elevated blood pressure (RR for highest vs lowest tertile, 0.91; 95% CI, 0.86-0.96).

**Table 2.** Time-Averaged Adjusted Risk of Elevated Blood Pressure by Tertiles of Census Tract Factors: Health and Retirement Study, 2006-2016

Tertiled census tract factor	Training set (n = 6472)		Test set (n = 6474)		Combined sample (N = 12 946)	
	RR (95% CI)^a^	*P* value^b^	RR (95% CI)^a^	*P* value^b^	RR (95% CI)^a^	*P* value^b^
**Proportion of owners who moved into tract after 1999**						
Lowest	1 [Reference]	NA	1 [Reference]	NA	1 [Reference]	NA
Middle	0.97 (0.90-1.05)	.49	0.95 (0.88-1.03)	.24	0.96 (0.91-1.01)	.14
Highest	0.90 (0.81-0.95)	.008	0.93 (0.85-1.00)	.05	0.91 (0.86-0.96)	.001
**Proportion of renters who moved into tract after 1999**						
Lowest	1 [Reference]	NA	1 [Reference]	NA	1 [Reference]	NA
Middle	0.95 (0.88-1.03)	.24	0.95 (0.88-1.03)	.20	0.95 (0.90-1.01)	.08
Highest	0.89 (0.82-0.96)	.004	0.97 (0.90-1.05)	.52	0.93 (0.88-0.98)	.01
**Proportion of housing units built before 1980**						
Lowest	1 [Reference]	NA	1 [Reference]	NA	1 [Reference]	NA
Middle	1.12 (1.03-1.22)	.006	1.04 (0.96-1.12)	.39	1.08 (1.01-1.14)	.01
Highest	1.12 (1.03-1.22)	.007	1.01 (0.931.10)	.80	1.06 (1.01-1.13)	.04

Abbreviations: NA, not applicable; RR, relative risk.

^a^ Estimates adjusted for 2006 vs 2008 baseline wave, log-transformed age in years, male sex, racial and ethnic designation (Hispanic or Latino, non-Hispanic Black, non-Hispanic White or other), US nativity, educational attainment (less than high school, high school degree or General Educational Development test achievement, 2-year college degree, at least a 4-year college degree), marital/partnership status (married/partnered, divorced/separated, widowed, never married/partnered), insurance coverage type (private, only federal/public, neither public or private), owns own home, household net income relative to Current Population Survey 2000 poverty line (above, below, not reported); weighted by probability of being eligible for study inclusion.

^b^ *P* value of continuous census tract variables adjusted for Simes correction for multiple comparisons.

In stratified analyses of this internally validated variable (Table 3), we observed nonsignificant differences by race and ethnicity. The inverse association between recent in-migration of homeowners appeared to be driven by non-Hispanic White and other older adults making up more than four-fifths of the sample (RR for highest vs lowest tertile, 0.91; 95% CI, 0.85-0.97). There were no associations for Hispanic or Latino participants (RR for highest vs lowest tertile, 0.84; 95% CI, 0.65-1.09; *P* = .78 for interaction of homeowners moved in after 1999 × Hispanic or Latino ethnicity) or non-Hispanic Black participants (RR for highest vs lowest tertile, 0.97; 95% CI, 0.85-1.11; *P* = .48 for interaction of homeowners moved in after 1999 × non-Hispanic Black race and ethnicity).

**Table 3.** Time-Averaged Adjusted Risk of Elevated Blood Pressure by Proportion of Homeowners Who Moved Into Census Tract After 1999, Stratified by Racial and Ethnic Designation: Health and Retirement Study, 2006-2016

Tertiled census tract factor	Non-Hispanic White or other (n = 10 676), RR (95% CI)^a^	Non-Hispanic Black (n = 1706), RR (95% CI)^a^	*P* value for interaction	Hispanic or Latino (n = 564), RR (95% CI)^a^	*P* value for interaction
Lowest	1 [Reference]	1 [Reference]	NA	1 [Reference]	NA
Middle	0.96 (0.90-1.02)	0.99 (0.88-1.12)	.83	0.96 (0.76-1.22)	.81
Highest	0.91 (0.85-0.97)	0.97 (0.85-1.11)	.48	0.84 (0.65-1.09)	.78

Abbreviations: NA, not applicable; RR, relative risk.

^a^ Estimates adjusted for 2006 vs 2008 baseline wave, log-transformed age in years, male sex, racial and ethnic designation (Hispanic or Latino, non-Hispanic Black, non-Hispanic White or other), US nativity, educational attainment (less than high school, high school degree or General Educational Development test achievement, 2-year college degree, at least a 4-year college degree), marital/partnership status (married/partnered, divorced/separated, widowed, never married/partnered), insurance coverage type (private, only federal/public, neither public or private), owns own home, household net income relative to Current Population Survey 2000 poverty line (above, below, not reported); weighted by probability of being eligible for study inclusion.

## Discussion

We performed one of the first untargeted evaluations of neighborhood factors associated with elevated blood pressure among a large, nationally representative sample of older adults. Across multiple comparisons of census tract factors and after adjustment for individual resources, our key finding was that relatively more recent in-migration of homeowners to a neighborhood appears to be modestly associated with a reduced likelihood of sphygmomanometer readings that reflect impaired blood pressure control among an older population. Though these patterns were apparent among non-Hispanic White and other participants, benefits among non-Hispanic Black and Hispanic or Latino adults may not be as consistent. Changes in neighborhood development and investment may differentially affect later-life blood pressure among diverse groups.

Recent in-migration of homeowners to a neighborhood may be a surrogate for the paradoxical influence that gentrification has on CVD risk factors among multiethnic neighborhoods. Observing more robust benefit among White than Black, Hispanic, or Latino older adults indicates how affluent new residents can contribute to local economy without benefitting existing marginalized communities.^19,20,36^ It is theorized that racial and ethnic minority residents of gentrifying neighborhoods may experience sociocultural exclusion due to the demographic as well as economic shifts that accompany an in-migration of neighbors wealthy enough to purchase property.^37,38^ As marginalized people are forced to relocate to more affordable areas and live with family members, gentrification may lead to a loss of social capital and community cohesion in former older-adult enclaves.^18^ The racial and ethnic minority individuals who remain may feel unwelcome and excluded from new amenities such as open spaces and food options with healthy choices that they cannot afford, instead coping with the increased stress with suboptimal health behaviors.^36,37^

Evaluating census tract variables in parallel while adjusting for multiple comparisons circumvents issues related to interpreting composite indices of area-level indices.^39^ Additionally, our study illustrates how not consistently accounting for collinear neighborhood characteristics and individual-level confounders may contribute to equivocal findings across studies.^40^ Area-level racial composition and income, which have the most extensive evidence base in neighborhood inequity research, were not independently associated with elevated blood pressure in our analysis. This runs counter to cross-sectional National Health and Nutrition Examination Survey findings, where non-poor White adults in neighborhoods that were predominantly White or above the poverty line had the lowest likelihood of hypertension.^4^ In comparison, our sample was older at baseline and had extensive available data on individual-level confounders such as home ownership and insurance status. As in our study, White-Black differences in hypertension prevalence were not observed among Medical Expenditure Panel Survey respondents in census tracts considered gentrifying based on relatively lower income, housing costs, or a lower percentage of college graduates.^5^ Estimates among the Hispanic or Latino subsample trended toward the same protective benefit on elevated blood pressure observed among non-Hispanic White and other participants. The small racial and ethnic minority subsamples in cohorts of older adults such as HRS grant researchers limited power to assess how neighborhood conditions contribute to disparities in CVD risk.

By explicitly evaluating co-occurring neighborhood conditions experienced by older adult communities, analyses such as ours can inform policy interventions that prioritize equity in the housing market and area development. Increasing older adult home ownership and affordable housing options are salient solutions for hypertensive disparities. Racial and ethnic minority and low-income people have been disproportionately underrepresented among adult homeowners aged 50 to 64 years since 1990; more than 80% percent of White households in this age group are homeowners, compared with fewer than 65% of Black and Hispanic households.^41^ Property is an asset that can equalize wealth among adults of retirement age, in order to facilitate CVD prevention.^16^ Rent stabilization, rental assistance programs, and federal standards for adequate housing can also allow marginalized adults to age in place, afford to engage in preventative health behaviors, and maintain their community networks.^42^

### Limitations

Consider our work in light of limitations common to cohort studies. Black, Hispanic, and Latino adults receive hypertension diagnoses and experience adverse CVD outcomes at earlier ages,^43^ contributing to differential selection by race and ethnicity into older adult research. Because HRS only has self-reported antihypertensive data among participants who report that they have high blood pressure, we could not ascertain whether a normotensive sphygmomanometer reading was due to initiating pharmacological treatment for more than one-third of our analytic sample. We only evaluated neighborhood characteristics at a single point in mid- to late life. Because the ACS began in 2005 and HRS is meant to be a representative sample of adults older than 50 years, our limited ability to ascertain neighborhood-level changes, individual-level residential history, and time-varying confounders at key life stages likely biases racial and ethnic differences toward the null.

While untargeted approaches such as ours can illuminate underexplored neighborhood exposures, evaluating numerous variables can produce chance findings; we attenuated this possibility by accounting for multiple comparisons and internally validating our results.^26,27^ We assessed local environmental conditions that are difficult to gauge with participant responses to psychosocial scales. However, administrative boundaries such as census tracts may neither accurately reflect how residents conceptualize their sense of neighborhood or correspond precisely with hypertensive risk. To protect participant privacy, HRS allows spatial linkages no more granular than the census tract level and prevents analyses that can identify participants via small samples. This renders us unable to delineate how accurately census tracts function as geographic units that identify excess risk of elevated blood pressure among participants. Because we cannot randomly assign participants to social determinants of health at the level of the individual or the census tract, positivity assumption violations pose near-intractable challenges when inferring causality between neighborhood context and CVD health.^44,45^ Participation trials that randomly assign evidence-based improvements to older adult communities are the natural next step in evaluating strategies that redress later-life blood pressure control disparities.

## Conclusions

In this cohort study of older adults, recent relocation of homeowners to a neighborhood was robustly associated with reduced likelihood of elevated blood pressure among White participants but not their racially and ethnically marginalized counterparts. Our findings indicate that gentrification may influence later-life blood pressure control. Future research should evaluate the mechanisms between neighborhood conditions over the life course and blood pressure control for vulnerable older adult communities.

## Notes

Supplement 1. **eFigure.** Selection of the study sample **eTable.** 2005-2009 American Community Survey census tract characteristics of Health and Retirement Study participants by hypertensive status, 2006-2008 (N=12946)

Supplement 2.
Data Sharing Statement
