# Supplementary material for: Neighborhood Characteristics and Elevated Blood Pressure in Older Adults
Source: JAMA Netw Open. 2023 Sep 25;6(9):e2335534. doi: 10.1001/jamanetworkopen.2023.35534 (PMC10520741; doi:10.1001/jamanetworkopen.2023.35534)
Supplement: Supplement 1. — eFigure. Selection of the study sample eTable. 2005-2009 American Community Survey census tract characteristics of Health and Retirement Study participants by hypertensive status, 2006-2008 (N=12946) [file jamanetwopen-e2335534-s001.pdf]

# Supplemental Online Content

Sims KD, Willis MD, Hystad PW, et al. Neighborhood characteristics and elevated blood pressure in older adults. *JAMA Netw Open*. 2023;6(9):e2335534. doi:10.1001/jamanetworkopen.2023.35534

**eFigure.** Selection of the study sample

**eTable.** 2005-2009 American Community Survey census tract characteristics of Health and Retirement Study participants by hypertensive status, 2006-2008 (N=12946)

This supplemental material has been provided by the authors to give readers additional information about their work.

© 2023 Sims KD et al. *JAMA Network Open.*

**eFigure.** Selection of the Study Sample

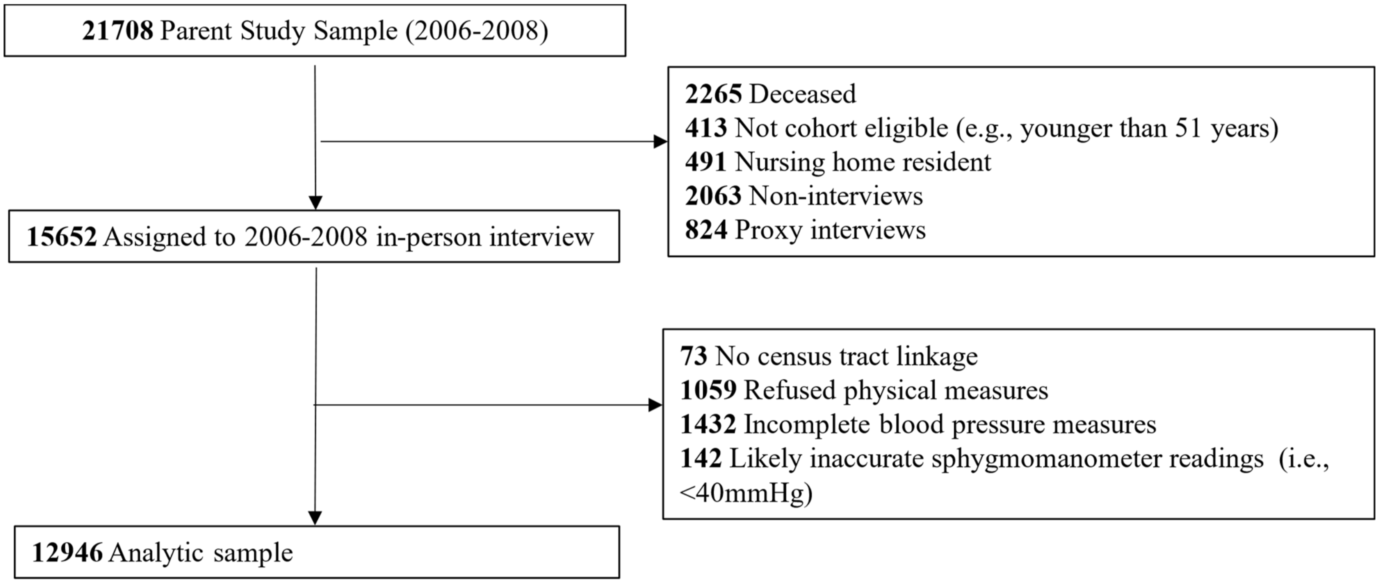


© 2023 Sims KD et al. *JAMA Network Open.*

**eTable.** 2005-2009 American Community Survey census tract characteristics of Health and Retirement Study participants by hypertensive status, 2006-2008 (N=12946)

| *Characteristic* | **Normotensive (N=8381)** | **Hypertensive (N=4565)** | ***p-value*** [a] |
|---|---|---|---|
| Unique census tracts, N | 4626 | 2395 | <0.001 |
| Population density per square mile[b] | 1978 (333, 4738) | 2128 (345, 4956) | 0.21 |
| Male, N (%) | 4107 (49) | 2237 (49) | 0.48 |
| *Age demographics* | | | |
| Aged Under 18, N (%) | 2011 (24) | 1096 (24) | 0.94 |
| Aged 18 to 64, N (%) | 5196 (62) | 2785 (61) | 0.18 |
| Aged 65 and over, N (%) | 1173 (14) | 685 (15) | 0.30 |
| *Racial/ethnic categories* | | | |
| Hispanic or Latino, N (%) | 1089 (13) | 593 (13) | 0.27 |
| Non-Hispanic Asian and Pacific Islander, N (%) | 335 (4) | 183 (4) | 0.50 |
| Non-Hispanic Black, N (%) | 838 (10) | 548 (12) | 0.001 |
| Non-Hispanic White, N (%) | 5950 (71) | 3150 (69) | <0.001 |
| Non-Hispanic Other or Multiple Races, N (%) | 168 (2) | 91 (2) | 0.56 |
| Non-U.S. nativity, N (%) | 838 (10) | 457 (10) | 0.57 |
| Speak only English at home, N (%) | 7040 (84) | 3835 (84) | 0.31 |
| Speak English well or better, N (%) | 8046 (96) | 4382 (96) | 0.24 |
| Linguistically isolated households, N (%) | 335 (4) | 183 (4) | 0.09 |
| *Educational attainment* | | | |
| No high school degree, N (%) | 1257 (15) | 685 (15) | 0.10 |
| High school degree, N (%) | 4861 (58) | 2693 (59) | 0.03 |
| College graduates, N (%) | 2263 (27) | 1187 (26) | 0.01 |
| Labor force participation rate, N (%) | 4448 (65) | 2922 (64) | 0.05 |
| Unemployed, N (%) | 587 (7) | 365 (8) | 0.04 |
| Employed in white collar occupations, N (%) | 2849 (34) | 1506 (33) | 0.05 |
| Employed in blue collar occupations, N (%) | 1006 (12) | 548 (12) | 0.11 |
| Drove alone to work, N (%) | 6537 (78) | 3561 (78) | 0.06 |
| Took public transportation to work, N (%) | 335 (4) | 183 (4) | 0.47 |
| Walked or bicycled to work, N (%) | 251 (3) | 137 (3) | 0.60 |
| Households with public assistance income, N (%) | 168 (2) | 91 (2) | 0.15 |
| Female-headed households with children, N (%) | 587 (7) | 320 (7) | 0.13 |
| Families with children in poverty, N (%) | 587 (7) | 365 (8) | 0.01 |
| In poverty, N (%) | 1089 (13) | 639 (14) | 0.02 |
| Aged 65 and over in poverty, N (%) | 754 (9) | 411 (9) | 0.07 |
| Living alone, N (%) | 838 (10) | 502 (11) | 0.21 |
| Aged 65 and over living alone, N (%) | 2179 (26) | 1233 (27) | 0.05 |
| Females aged 65 and over living alone, N (%) | 335 (4) | 228 (5) | 0.06 |
| Homeowners, N (%) | 5950 (71) | 3196 (70) | 0.04 |

© 2023 Sims KD et al. *JAMA Network Open.*

| *Characteristic* | **Normotensive (N=8381)** | **Hypertensive (N=4565)** | ***p-value*** [a] |
|---|---|---|---|
| Vacant homes, N (%) | 838 (10) | 457 (10) | 0.51 |
| Vacant rentals, N (%) | 587 (7) | 320 (7) | 0.07 |
| Vacant units for sale, N (%) | 168 (2) | 91 (2) | 0.30 |
| Single unit home, N (%) | 5615 (67) | 3013 (66) | 0.05 |
| More than one person per bedroom, N (%) | 251 (3) | 137 (3) | 0.07 |
| *Housing unit construction* | | | |
| Units built before 1980, N (%) | 5029 (60) | 2830 (62) | <0.001 |
| Units built between 1980 and 1999, N (%) | 2430 (29) | 1278 (28) | <0.001 |
| Units built after 1999, N (%) | 922 (11) | 457 (10) | 0.03 |
| Homeowners moved after 1999, N (%) | 3436 (41) | 1826 (40) | 0.001 |
| Homeowners moved after 2004, N (%) | 1257 (15) | 685 (15) | 0.20 |
| Renters moved after 1999, N (%) | 6872 (82) | 3698 (81) | 0.001 |
| Renters moved after 2004, N (%) | 4358 (52) | 2328 (51) | 0.19 |
| Median home value, $1000s (IQR)[bc] | 190 (122, 338) | 186 (107, 320) | 0.24 |
| Median rent (IQR)[bc] | 994 (731, 1208) | 916 (723, 1166) | 0.004 |
| Median household income, $1000s (IQR)[bc] | 56 (41, 76) | 55 (43, 72) | <0.001 |

[a]p-value (Pearson chi-squared test for categorical variables, Wilcoxon rank sum test for continuous variables) for difference between normotensive versus hypertensive sphygmomanometer reading
[b]Values are expressed as median (interquartile range).
[c]All US dollar values are adjusted for inflation to 2018 values using the Consumer Price Index Research Series Using Current Methods as developed by the Bureau of Labor Statistics. The estimated median household income, house value, and gross rent values are calculated from adjusted and aggregated count data to derive interpolated medians.

© 2023 Sims KD et al. *JAMA Network Open.*
