# Supplementary material for: Neighborhood Characteristics and Elevated Blood Pressure in Older Adults
Source: JAMA Netw Open. 2023 Sep 25;6(9):e2335534. doi: 10.1001/jamanetworkopen.2023.35534 (PMC10520741; doi:10.1001/jamanetworkopen.2023.35534)
Supplement: Supplement 2. — Data Sharing Statement [file jamanetwopen-e2335534-s002.pdf]

Data Sharing Statement

Sims. Neighborhood Characteristics and Elevated Blood Pressure in Older Adults. *JAMA Netw Open*. Published September 25, 2023. doi:10.1001/jamanetworkopen.2023.35534

Data
Data available: No

Additional Information
Explanation for why data not available: The Health and Retirement Study provides access to individual-level data for registered users, and offers access to area-level data via a remote data enclave after completion of an application.
